# The association between preoperative anemia, blood transfusion need, and postoperative complications in adult cardiac surgery, a single center contemporary experience

**DOI:** 10.1186/s13019-023-02132-5

**Published:** 2023-01-07

**Authors:** Hani Mufti, Faisal Alsharm, Mohanad Bahawi, Mohammed Almazmumi, Yazeed Alshaikh, Amir Abushouk, Abdullah Algarni, Sahal Jamalallail, Mohammed Almohammadi

**Affiliations:** 1 Division of Cardiac Surgery, Department of Cardiac Sciences, King Faisal Cardiac Center, King Abdulaziz Medical City, Ministry of National Guard Health Affairs, Jeddah, Saudi Arabia; 2 King Abdullah International Medical Research Center, Ministry of National Guard Health Affairs, Jeddah, Saudi Arabia; 3 College of Medicine, King Saud Bin Abdulaziz University for Health Sciences, Ministry of National Guard Health Affairs, Jeddah, Saudi Arabia; 4 Department of Pathology and Laboratory Medicine, King Abdulaziz Medical City, Ministry of National Guard Health Affairs, Jeddah, Saudi Arabia; 5 Division of Cardiac Surgery, Department of Cardiac Sciences, King Faisal Cardiac Center, King Abdulaziz Medical City, Ministry of National Guard Health Affairs, P.O. Box 9515, Mail Code: 6599, Jeddah, 21423 Saudi Arabia

**Keywords:** Cardiac Surgery, Anemia, Transfusion, Complications

## Abstract

**Background:**

The impact of preoperative anemia on postcardiac surgery outcomes is an area of great debate. Although several large-scale studies have been conducted, they have demonstrated conflicting results. A limited number of studies have been conducted in the Middle East. The primary aim of this study was to investigate the association between preoperative anemia and the need for blood transfusions, as well as major postoperative complications.

**Methods:**

Adult patients who underwent cardiac surgery at King Faisal Cardiac Center in Jeddah between June 2016 and January 2020 were included in this retrospective cohort study. The study excluded patients with hereditary preoperative anemia. Among the variables studied were variables related to demographics, comorbidities, laboratory data, operation-related data, in-hospital complications, and mortality.

**Results:**

The mean preoperative hemoglobin (Hb) level was 13.2 g/dL (SD ± 1.8). The overall mortality rate was 4.6%. A lower preoperative Hb level (*p* value = 0.016), postoperative day 1 WBC count (*p*-value = 0.003), and prolonged cross clamp time (*p* value < 0.001) were significantly associated with mortality. A lower Hb level during the preoperative period or within the first three days of surgery was associated with a higher blood transfusion requirement. However, there was no significant association between blood transfusion and postoperative complications. A multivariate stepwise logistic regression model was developed and several pre and intra operative factors were predictive of the need PRBCs transfusion after cardiac surgery (which included: older age, female gender, lower pre-operative hemoglobin and longer cardio-pulmonary bypass time), with had a predictive accuracy of around ~ 86%.

**Conclusion:**

Based on our single center study, patients with preoperative lower Hb levels are at higher risk of mortality. However, blood transfusion does not seem to increase the risk of postoperative complications. Optimal utilization of blood products is an important quality metric and identification of patients at higher risk of requiring PRBCs transfusion prior to cardiac surgery can help in implementing pre or intra operative strategies to minimize the need for transfusion.

## Introduction

According to the World Health Organization (WHO), anemia is defined as a hemoglobin (Hb) level of less than 13.0 g/dl in adult males or less than 12.0 g/dl in nonpregnant adult females [1]. Preoperative anemia may be associated with the worst postoperative outcomes in cardiac surgery, with an estimated prevalence ranging from 16 to 54% [2–5]. A multicenter study performed by Sallam et al. demonstrated that patients with preoperative anemia are more susceptible to developing sternal wound infections than nonanemic patients [3]. Another study showed that the one-year mortality rate is significantly higher in patients with preoperative anemia, but there were no significant differences in one-month mortality rates [6]. Nevertheless, other studies suggested that preoperative anemia is not an independent risk factor after multivariate-adjusted analysis but rather a marker of underlying comorbidities, including age, acute coronary syndrome, diabetes, renal insufficiency, and extensive coronary disease, which are associated with poor postoperative outcome [7, 8].

Furthermore, preoperative anemia is an independent predictor for increased transfusion requirements during cardiac surgery [9, 10]. A study performed by Nguyen et al. showed that the transfusion rate in cardiac surgery among anemic patients was 53% versus 10% in the nonanemic group [11]. Transfusion of blood and blood products is associated with the risk of posttransfusion complications, and therefore, anemic patients are at higher risk of developing postoperative complications [12, 13]. The most common postoperative complications found were mortality, organ dysfunction, serious infections, renal failure, cardiac complications, neurologic complications, overall morbidity, and prolonged ventilator support [12, 14]. Al-harbi et al. found a significant increase in the risk of infection after coronary artery bypass grafting (CABG) in patients who received blood transfusions, and pneumonia was the most reported infection after CABG [12]. In addition, patients who received a blood transfusion had longer preoperative and postoperative hospitalization times than those who did not receive any blood transfusions [12]. A longer hospitalization duration predisposed the patients to a higher risk of developing nosocomial infections [12]. Other studies, however, have found no association between blood transfusion and the incidence of infection, acute kidney injury (AKI), or long-term mortality (30 days to 5 years) [15–17].

Transfusing blood may be necessary during cardiac surgery. As a result, it is crucial to determine whether blood transfusion is significantly related to postoperative complications in cardiac surgery. A higher number of transfused units is associated with an increased risk of morbidity and mortality in cardiac surgeries [18]. According to two cohort studies, postoperative complications such as infection are associated with transfusion in a dose-dependent pattern, and the studies concluded that the risk of infection was increased by 20% with each unit of blood given postoperatively. However, the exact threshold of transfusion units was not identified [12, 14]. Another study suggested that the transfusion of blood products may have no harmful effect if the total transfused units were less than 5.5 units in cardiac surgery patients [19]. Although the “Restrictive or Liberal Red-Cell Transfusion for Cardiac Surgery Randomized Controlled trial” has addressed the issue of restrictive versus liberal transfusion strategy [20], we assumed that a preoperative hemoglobin level of 10 mg/dl in patients with ischemic heart disease might still be a trigger for transfusion.

The lack of recent local studies and the contradiction between the results of existing studies, with regard to whether preoperative anemia is associated with the need for transfusion of blood and blood products, as well as the postoperative outcomes in cardiac surgery, necessitate further investigation.

## Methods

This is a retrospective cohort study that included 386 adult patients (over the age of 14) who underwent cardiac surgery at the King Faisal Cardiac Center (KFCC) in Jeddah between June 2016 and January 2020. All types of cardiac surgery were included, such as CABG, valve repair, and valve replacement. The study excluded all patients with genetic or hereditary preoperative anemias.

The data were collected from the medical records department using the BESTCARE system and the laboratory and pathology information system after Institutional Review Board (IRB) approval. Throughout the research process, all ethical considerations were followed. We defined anemia according to the WHO definition of a hemoglobin level of less than 13 g/dl for men and less than 12 g/dl for nonpregnant women. In addition, complications were collected during surgery and through the next 30 days after surgery during the follow-up period.

### Primary and secondary end points

The primary objectives of this study were 1) to identify the effect of preoperative anemia as a predictor of postoperative outcomes and 2) to assess the relationship between anemia and the necessity of blood resource utilization after cardiac surgery. The secondary objectives were 1) to evaluate transfusion-related complications after cardiac surgery; 2) to identify the effect of the blood and blood product unit age on postoperative outcomes; and 3) to identify the prevalence of preoperative anemia in patients who underwent cardiac surgery.

### Variables of interest

The following variables categories were extracted from the electronic medical records: Demographics (Age, date of birth, Gender), Comorbidities (Ischemic Heart Disease, Diabetes Mellites, Hypertension, Dyslipidemia, Chronic Kidney Disease, Pre-operative Dialysis, Smoking, Chronic Obstructive Pulmonary Disease, Stroke, Other Comorbidities), Pre-Operative laboratory investigations within 1 week from surgery (Hemoglobin, Hematocrit, Mean corpuscular volume, Mean corpuscular hemoglobin, Platelets, White Blood cell count, Creatinine, estimated Glomerular Filtration rate, Hemoglobin A1C), Operation Related (cardiac diagnosis, Procedure Status, Procedure performed, Cross Clamp Time, Cardio-pulmonary Bypass Time), Dates (Date of admission, Date of Surgery, Date of Discharge from ICU, Date of Discharge from Hospital, Length of stay in ICU, Length of stay in the Hospital), Post Operative day 1, 3 and at discharge: Hemoglobin, Hematocrit, Mean corpuscular volume, Mean corpuscular hemoglobin, Platelets, White Blood cell count, Creatinine need for Blood or blood products transfusion, date of blood transfusion), In-hospital Complications (Infection type (Pneumonia, urinary tract infection, Sepsis, Wounds), Stroke, Seizures, Delirium, Renal Failure, Dialysis, Surgical Bleeding, Tamponade, Return to the operating room, Arrhythmia, need for permanent pacemaker, need for coronary cardiac intervention, Mortality within 30 days from surgery). Post operative Infection indicates any infection that is diagnosed with either a positive blood or body fluid culture with clinical or laboratory evidence of infection (fever, increase in white blood cells, increase of inflammatory markers or hemodynamic compromise as an evidence of septic shock).

### Statistical analysis

All statistical analyses were performed using R software, version 4.1.1 (R Foundation for Statistical Computing, Vienna, Austria). We compared the features of adult patients who underwent cardiac surgery and received blood product transfusions to those of the patients who did not. The mean and standard deviation were used for continuous variables that had a normal distribution and were compared using the two-sample t test or Welch two-sample t test if the two groups had unequal variance. Continuous variables that were not normally distributed were reported using the median and interquartile range and were compared using the Wilcoxon rank-sum test. To confirm whether a continuous variable came from a normal distribution, we applied the Shapiro‒Wilk test of normality, histograms, and quantile‒quantile plots. Categorical variables were reported as frequencies and percentages and were analyzed by Chi-square or Fisher’s exact test as appropriate. The Kruskal‒Wallis test was used for ordinal attributes. To identify pre and intra operative risk factors for the need for post-operative packed red blood cell (PRBC) transfusion after cardiac surgery, univariate analysis using logistic regression was used to identify significant pre and intra-operative predictors with a *p*-value less than 0.1. All significant predictors from the univariate analysis were included in a multivariate analysis using logistic regression. To further identify important predictors, stepwise logistic regression analysis was used to exclude non-significant predictors. All statistical tests were two-tailed, and *p* values < 0.05 were considered significant.

## Results

### General characteristics

Between January 2016 and January 2020, 386 adult patients underwent open heart surgery. The mean age at operation was 58 years (standard deviation (SD) ± 13), and 71% of the patients were males (see Table 1). The most performed procedure was CABG (67.2%), and the most common cardiac diagnosis was ischemic heart disease (68.4%). The prevalence of anemia in our cohort, in the diagnosis was based on the WHO definition of anemia (1), was 34.3%, with a 32.9% and 37.7% prevalence in males and females, respectively. All patients had Anti-platelets (Aspirin, Clopidogrel or Ticagrelor) stopped at least 3–5 days prior to elective or semi-urgent surgery. Only 6 patients underwent emergency surgery (1.6%), and all underwent CABG. Thirty-nine patients (~ 10% of the cohort) underwent redo sternotomy and cell saver was prepared in all redo sternotomy cases but no processed blood was not used in any of these cases. It is not part of our practice to use pre-operative autologous donation, acute nonmonomeric hemodilution or auto transfusion of chest drains blood due to institutional regulations and other technical limitations.

**Table 1:** General characteristics

Variable	Overall
Number of patients	386
Age in Years (mean (SD))	58 (13)
Male Gender (%)	275 (71.2)
Height in cm (mean (SD))	163.6 (8.6)
BSA (mean (SD))	2 (1.8)
Weight in kg (mean (SD))	78 (15)
BMI (mean (SD))	29 (6)
IHD (%)	301 (78)
DM (%)	255 (66)
HTN (%)	290 (75)
DLP (%)	250 (65)
CKD (%)	56 (14.5)
PreOp Dialysis (%)	14 (3.6)
Smoking (%)	80 (20.7)
COPD (%)	11 (2.8)
PreOp Stroke (%)	31 (8.0)
Length of Stay in Hospital in Days (mean (SD))	16.9 (11.8)
CPB in minutes (mean (SD))	123.3 (68.9)
Cross Clamp in minutes (mean (SD))	83.3 (52.9)

*BMI* Body Mass Index, *BSA* Body Surface Area, *CKD* Chronic Kidney Disease, *COPD* Chronic Obstructive Pulmonary Disease, *CPB* Cardio-Pulmonary Bypass, *DLP* Dyslipidemia, *DM* Diabetes Mellites, *IHD* Ischemic Heart Disease, *PreOp* Preoperative

### Mortality in relation to pre- and postoperative labs

The overall mortality was 4.6%. The preoperative blood work revealed that the mean Hb level was 13.2 g/dl (SD ± 1.8), and almost 93% of the patients had a Hb level above 10 g/dl. The mean postoperative Hb levels were 10.3, 9.4, and 10.5 at day 1 after surgery, day 3 after surgery and at discharge, respectively (*p* value =  < 0.05). The factors that were significantly associated with mortality were as follows: 1- lower preoperative Hb level (*p* value = 0.016), 2- higher white blood cell (WBC) count at day 1 after surgery (*p* value = 0.003) and at discharge day (*p* value < 0.001), and 3- prolonged cross clamp time (*p* value < 0.001) (see Table 2).

**Table 2:** Mortality relationship to blood work at different times from surgery

	Hemoglobin			Platelets			WBC			Creatinine		
	Mortality			Mortality			Mortality			Mortality		
	No = 363 (93.3%)	Yes = 18 (4.7%)	*P*-value	No = 363 (93.3%)	Yes = 18 (4.7%)	*P*-value	No = 363 (93.3%)	Yes = 18 (4.7%)	*P*-value	No = 363 (93.3%)	Yes = 18 (4.7%)	*P*-value
PreOp (mean (SD))	13.2 (1.8)	12.1 (1.8)	0.016*	253.8 (77.8)	229.4 (86.8)	0.208	8.4 (11.7)	8.3 (2.3)	0.992	108.3 (123.4)	102.5 (36.3)	0.849
PostOp D1 (mean (SD))	10.3 (1.2)	9.5 (2.1)	0.064	206.3 (64.3)	174.5 (69.3)	0.082	13.9 (4.1)	17.4 (6.5)	0.003*	107.3 (111)	147.9 (92.6)	0.212
PostOp D3 (mean (SD))	9.4 (1.1)	9.5 (1.2)	0.876	188.4 (67.9)	115.4 (62.5)	< 0.001*	13.4 (14.7)	14.2 (5)	0.845	105.8 (121.6)	134.2 (70.4)	0.442
At Discharge (mean (SD))	10.5 (1.1)	9.2 (1.3)	< 0.001*	403.4 (147.5)	267.5 (191.6)	0.005*	10.1 (2.9)	16.1 (9.9)	< 0.001*	99.3 (99)	161.3 (85.1)	0.051

The asterisk (*) indicates a p-value < 0.05

*D1* Day 1, *D3* Day 3, *Preop* Preoperative, *PostOp* Postoperative, *WBC* White Blood Cells count

### Blood product transfusion

**Fig. 1:**
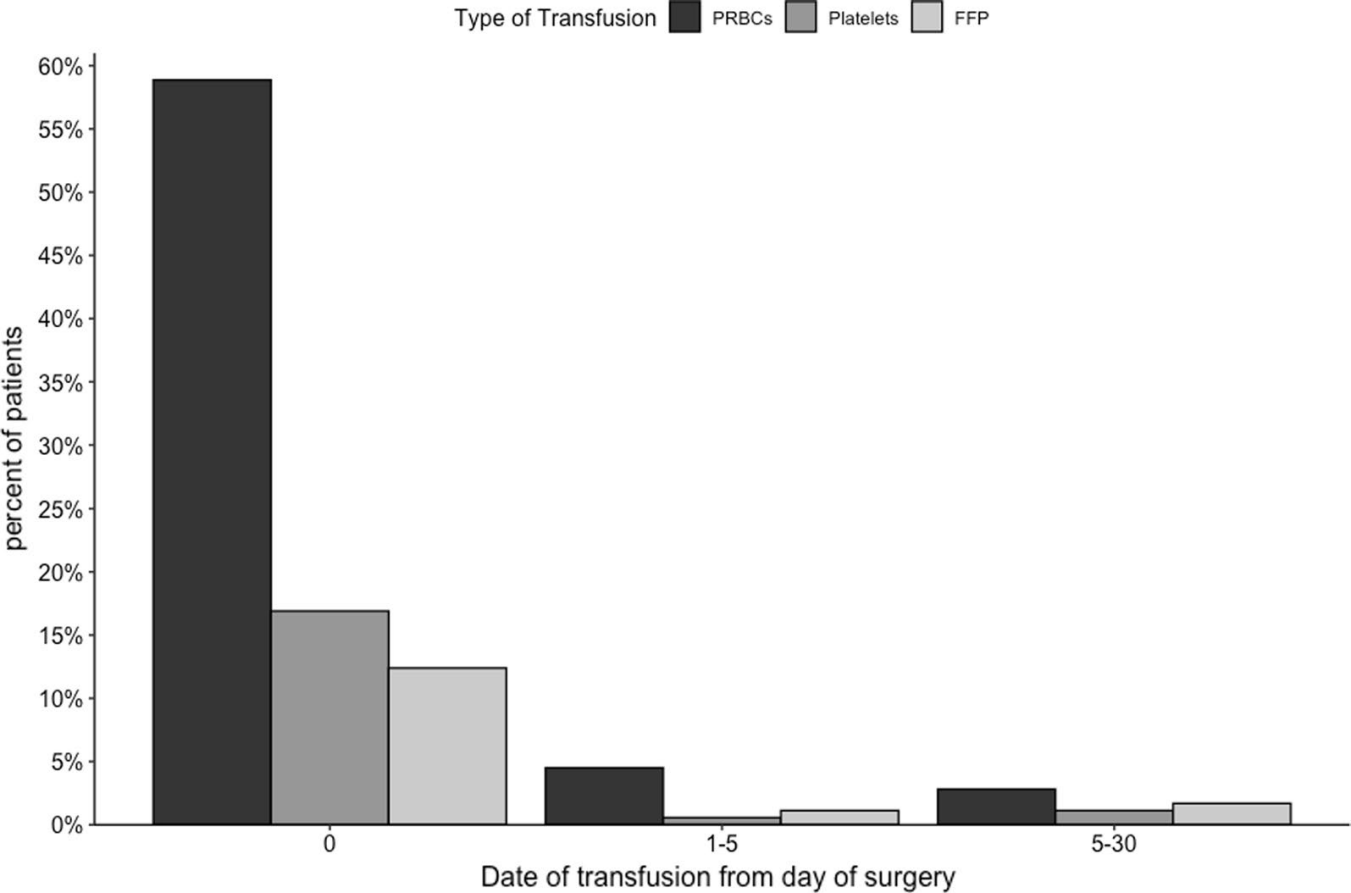
Date of transfusion from day of surgery. PRBCs: Packed Red Blood Cells, FFP: Fresh Frozen Plasma. The above figure highlights that most patients (almost 60%) will receive blood products within the first 24 h from surgery

**Fig. 2:**
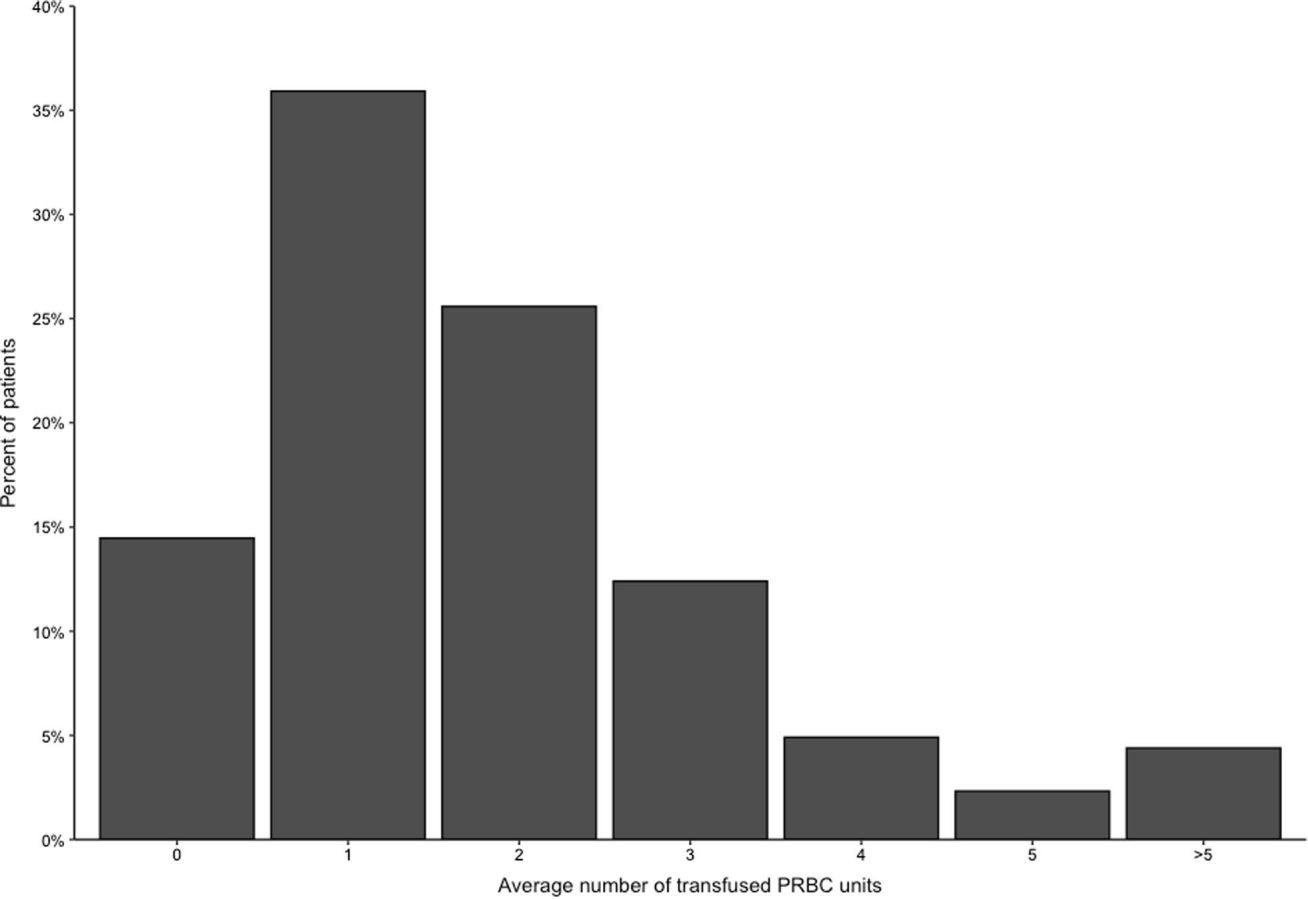
Average number of transfused PRBC units. PRBC: Packed Red Blood Cells. The above figure demonstrates that most patients (~ 50%) will receive only 1 or 2 units and less than 5% of our cohort required more than 5 units of PRBCs

**Fig. 3:**
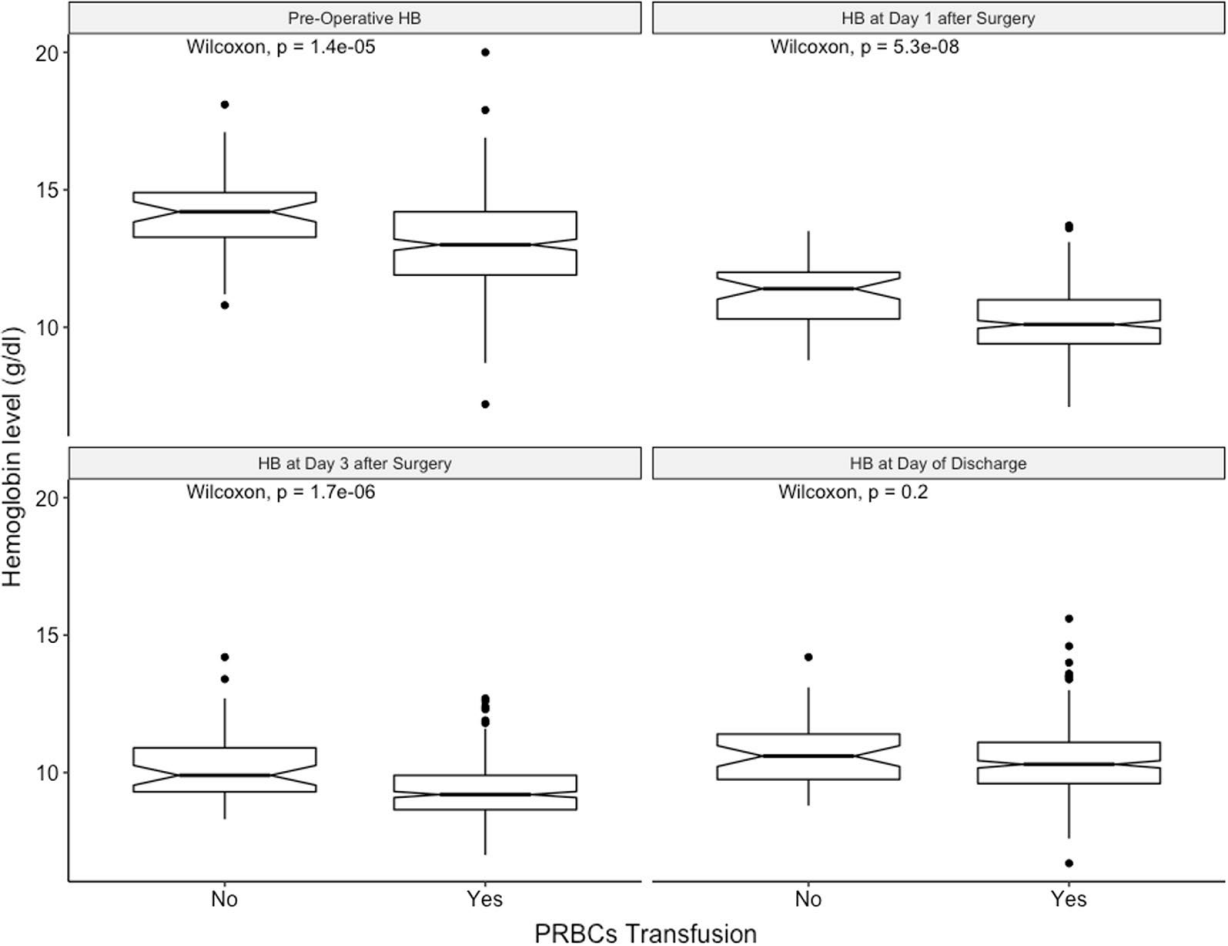
Pre-operative HB level and its relationship PRBCs Transfusion at different time frames from surgery. PRBCs: Packed Red Blood Cells, HB: Hemoglobin, g/dl = Gram per Deciliter. The above figure demonstrates the difference of Hemoglobin level between the patients who received blood transfusion versus the ones who did not. Patients who received PRBCs transfusion had much lower pre-operative Hb compared to the ones who did not (13 vs 14.2, *p*-value =  < 0.001). The same applies for Day 1 and day 3 after surgery with patients having Hb levels 10 g/dl or lower after surgery are requiring PRBCs transfusion (both having a *p*-value < 0.001). However, at discharge there was difference most likely due to that most patients had a Hb level above 10 g/dl

The rate of blood product transfusion was high in our cohort, with PRBCs being the main product that was transfused (85.5%), followed by platelets (24.1%), and finally fresh frozen plasma (18.9%) (see Table 3). Most patients received blood products on the day of surgery in the intensive care unit (day 0) (see Fig. 1). Based on the “Restrictive or Liberal Red-Cell Transfusion for Cardiac Surgery Randomized Controlled Trial” (20), we assumed that having a preoperative Hb level below 10 g/dl was a predictor of the need for transfusion. However, this was not the case in our cohort (*p* value = 0.0899), and most patients in our cohort had a preoperative Hb level above 10 g/dl (95.1%). Most patients in our cohort received 2 or fewer units of PRBCs (61.5%) (see Fig. 2). When we examined the association of Hb level with the requirements for PRBC transfusion, we discovered that the lower the Hb level at either the preoperative period or within the first 3 days of surgery, the more likely that patients will require blood transfusion (see Fig. 3). Although blood and blood product transfusions are usually associated with postoperative complications (especially infectious complications), this was not evident in our cohort (see Table 4). It is worth noting that we were not able to evaluate the relationship between amount of bleeding in the operating room and need for blood products transfusion because this was not captured objectively in the patient chart.

**Table 3:** Number of patients receiving specific number of Blood products units transfused in the study cohort

Number of units	Number of patients who received (% out of 386 patients)		
	PRBCs	Platelets	FFP
0	56 (14.5%)	337 (87.3%)	313 (81.1%)
1	139 (36%)	1 (0.3%)	2 (0.5%)
2	99 (25.7%)	2 (0.5%)	12 (3.1%)
3	47 (12.2%)	6 (1.6%)	36 (9.3%)
4	19 (4.9%)	22 (5.7%)	9 (2.3%)
5	9 (2.3%)	4 (1.04%)	8 (2.1%)
6	6 (1.6%)	12 (3.1%)	8 (2.1%)
> 6	11 (2.9%)	2 (0.5%)	6 (1.6%)

*PRBCs* Packed Red Blood Cells, *FFP* Fresh Frozen Plasma, *n* number

**Table 4:** PRBC transfusion and complications

	Level	Overall	PRBCs transfusion		*p* value
			No	Yes	
Number of patients		387	51	331	
PostOp Infection Pneumonia (%)	No	307 (79.3)	43 (84.3)	264 (79.8)	0.739
	Yes	58 (15.0)	6 (11.8)	52 (15.7)	
	NA	22 (5.7)	2 (3.9)	15 (4.5)	
PostOp Infection UTI (%)	No	323 (83.5)	47 (92.2)	276 (83.4)	0.21
	Yes	42 (10.9)	2 (3.9)	40 (12.1)	
	NA	22 (5.7)	2 (3.9)	15 (4.5)	
PostOp Infection Wound Infection (%)	No	273 (70.5)	40 (78.4)	233 (70.4)	0.491
	Yes	87 (22.5)	9 (17.6)	78 (23.6)	
	NA	27 (7.0)	2 (3.9)	20 (6.0)	
Mortality (%)	No	352 (91.0)	49 (96.1)	303 (91.5)	0.225
	Yes	18 (4.7)	0 (0.0)	18 (5.4)	
	NA	17 (4.4)	2 (3.9)	10 (3.0)	

*PostOp* Postoperative, *PRBCs* Packed Red Blood Cells, *UTI* Urinary Tract Infection

### Pre and Intra operative predictors of need for PRBCs transfusion after cardiac surgery

**Fig. 4:**
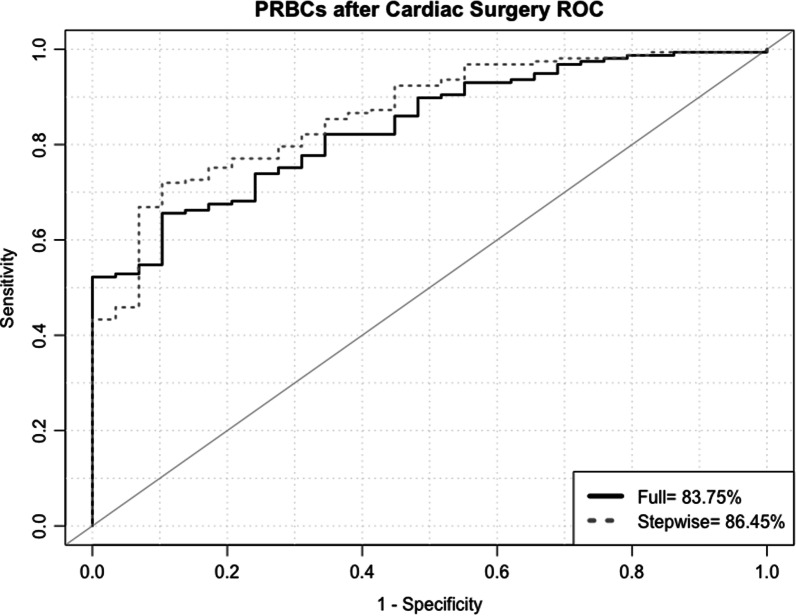
PRBCs after Cardiac Surgery Receiver Operator Curve. PRBCs: Packed Red Blood Cells, ROC: Receiver Operator Curve. The above figure highlights that the stepwise logistic regression model had a better area under the receiver operator curve with higher sensitivity and reasonable specificity

Using logistic regression modeling, all pre and intra operative variables were examined using bivariate analysis. Five variables were identified as significant with a *p*-value < 0.1 (These are: Age in Years, Female Gender, DM, Pre-operative Hemoglobin level and Cardio-pulmonary bypass time in minutes). All these variables were included in a multivariate logistic regression. The full multivariate logistic regression that included all 5 variables had a Receiver Operator Curve Area Under the Curve (ROC-AUC) of 83.75% (95% Confidence interval = 77.1–90.4%). We used stepwise logistic regression to eliminate un-necessary variables and generate a parsimonious model, which eliminated DM from the model and improved the model general performance by a significant increase in the model ROC-AUC to 86.45 (95% Confidence interval = 80.4–92.86%). (See Table 5 and Fig. 4).

**Table 5:** Univariate and Multivariate pre and intra operative Predictors of PRBCs Transfusion after Cardiac Surgery in Adults

Variable	Units	Univariate			Full Model			Stepwise		
		OR	95% CI	*p*-value	OR	95% CI	*p*-value	OR	95% CI	*p*-value
Age in Years		1.02	[1.00–1.04]	0.060	1.05	[1.00–1.09]	0.037	1.05	[1.01–1.09]	0.025
BMI in KG/m^2^		0.97	[0.92–1.02]	0.278	NA	–	–	NA	–	–
Gender	Male	Ref			Ref			Ref		
	Female	3.45	[1.43–8.36]	0.006	3.52	[0.79–15.7]	0.099	3.5	[0.8–15.3]	0.096
DM	No	Ref			Ref			NA	–	–
	Yes	2.16	[1.18–3.93]	0.012	1.02	[0.35–2.96]	0.968	NA	–	–
IHD	No	Ref			NA	–	–	NA	–	–
	Yes	1.15	[0.57–2.32]	0.695	NA	–	–	NA	–	–
COPD	No	Ref			NA	–	–	NA	–	–
	Yes	1.56	[0.19–12.42]	0.677	NA	–	–	NA	–	–
Pre Operative Hemoglobin level in gm/dl		0.68	[0.57–0.82]	0.001	0.62	[0.45–0.82]	0.003	0.61	[0.45–0.84]	0.003
Procedure is isolated CABG	Yes	Ref			NA	–	–	NA	–	–
	Other	1.15	[0.6–2.2]	0.671	NA	–	–	NA	–	–
CPB in minutes		1.01	[1.00–1.02]	0.015	1.02	[1.01–1.03]	0.002	1.02	[1.01–1.03]	0.002

*BMI* Body Mass Index, *DM* Diabetes Mellites, *IHD* Ischemic Heart Disease, *COPD* Chronic Obstructive Pulmonary Disease, *CABG* Coronary Arteries Bypass Grafts Surgery, *CPB* Cardio-Pulmonary Bypass Time, *OR* Odds ratio, *CI* Confidence Interval

## Discussion

Preoperative anemia is common among cardiac surgery patients, with a prevalence ranging from 16 to 54% (2). Preoperative anemia is still not a definitive parameter to consider when predicting postoperative outcomes. This study aimed to identify the effect of preoperative anemia as a predictor for postoperative outcomes. Additionally, this study investigated whether preoperative anemia was associated with an increased need for blood transfusion and whether the transfusion of blood and blood products was associated with worse postoperative outcomes.

The results of the analysis indicate that a lower preoperative Hb level was significantly associated with increased mortality rates, suggesting that preoperative anemia is an independent risk factor for mortality. Preoperative anemia-associated complications may be attributed to reduced perfusion and oxygen delivery to tissues resulting in organ dysfunction. These findings were consistent with those of Miceli et al., who found a threefold increase in mortality in a study of 7738 patients [21]. Similar results were found by Pala et al. using hematocrit as a parameter for anemia instead of hemoglobin levels [22]. Therefore, the assessment and correction of anemia before surgery may reduce mortality.

Additionally, higher levels of WBCs at postoperative day 1 and longer cross clamp time were also associated with higher mortality rates. Ascione et al. observed that WBC levels peaked 36 to 60 h after cardiopulmonary bypass [23]. An exaggerated inflammatory response by WBCs could lead to adverse outcomes especially in cardiac surgery patients with the use of cardio-pulmonary bypass leading to an exaugurated systemic inflammatory response syndrome in a substrate of patients with specific risk factors (example: age, bleeding, blood transfusion, prolonged cross clamp or pump time, hypothermia, elevated pre-operative WBC count…etc.) [24–26]. In addition, a similar result was depicted by Al-sarraf et al., who found a significant association of prolonged cross clamp time with morbidity and mortality in a study of 3799 patients who underwent cardiac surgery [27].

Furthermore, although we found that having a preoperative Hb level below 10 g/dl was not associated with the need for blood product transfusion, lower Hb levels during the preoperative period and the first three days after surgery were associated with an increased need for PRBC transfusion. The reduced oxygen delivery caused by anemia may increase the requirement of transfusion as the body attempts to correct for the deficient end organ tissue perfusion. These results were consistent with what Koch et al. observed in a study of 11963 patients, which found that older age, lower body mass index (BMI), measures of renal dysfunction, and lower preoperative hematocrit levels, which are other parameters for measuring anemia, are predictors for blood transfusion requirements [28].

The analysis of the data showed that transfusion of blood and blood products had no significant association with transfusion-related complications, including pneumonia, urinary tract infection (UTI), wound infection, and mortality, after cardiac surgery. These results contrasted with the results of a 459-patient study conducted in Riyadh by Al-Harbi et al., which found that there was a significantly increased risk of postoperative infection and transfusion, and the study demonstrated a dose-related association [12]. Additionally, Koch et al. also found a dose-dependent relationship between the number of PRBC transfusions and worse postoperative outcomes, such as in-hospital mortality, serious infection, neurological complications, renal failure, overall morbidity, and prolonged ventilatory support. This relationship continued to be strong even after adjusting for the risk factors associated with worse postoperative outcomes after CABG [28]. A study performed by Leal-Noval et al. found that receiving 4 or more units of blood components was associated with serious postoperative outcomes and mortality [29]. Most of the patients in our study received 2 units or less (see Fig. 1). This may explain why slightly higher rates of postoperative infections were observed among transfused patients, although this was statistically nonsignificant.

The association between preoperative anemia and other postoperative outcomes, including infections, arrhythmias, neurological complications, renal complications, surgical complications, permanent pacemaker requirement, coronary cardiac intervention requirement, and length of hospitalization, needs to be addressed in future studies to clarify the role of anemia in cardiac surgery complications. The utilization of blood products is an important quality metric, especially after cardiac surgery, and is associated with positive outcomes. Medical teams managing cardiac surgery patients should strive to implement several preventive strategies to minimize the need for blood products transfusion [30]. Based on our findings, out of all pre and intra-operative variables: older age, lower pre-operative hemoglobin level and longer time on bypass were a strong predictors for the need of PRBCs transfusion after cardiac surgery in multivariate logistic modeling. Although lower pre-operative hemoglobin level might be associated with chronic diseases and is a function of age, this model can be used to predict patients at higher risk of requiring transfusion and optimize their medical status prior to surgery to minimize the amount of transfusion and its associated complications.

### Limitations

There are some limitations to this study that should be acknowledged. First, retrospective studies are prone to data extraction errors from medical records. Second, patients with paper files were excluded because their data were missing, illegible, or unclear. Third, because the center employs multiple cardiac surgeons, standardization of the results can be challenging. Fourth, this study was conducted at a tertiary hospital, which may indicate that its patients are in a more critical situation. Fifth, we were not able to establish the relationship between the blood product age (duration of storage of the blood and blood products) and postoperative outcomes, as the data were not available. Sixth, the amount of bleeding in the operating room and after was not captured in our study, which might influence the amount of blood transfusion.

## Conclusion

The primary aim of our single center study was to identify if there is an association between Hb level and complications, of which we only found an association with mortality. Further studies analyzing the association between preoperative anemia and each of the other major postoperative complications are needed. Future work investigating the isolated impact of postoperative WBC count on complications is warranted. Lower preoperative Hb levels appear to increase the requirements for transfusion, predisposing patients to possible complications. Nevertheless, in our study, blood and blood product transfusion seemed to have no significant impact on postoperative complications. Older age, lower pre-operative hemoglobin and longer bypass time were highly predictive for the need for PRBCs after cardiac surgery. The classification of blood products based on the storage duration and method of storage are also suggested in future studies for better elimination of a possible confounder.

## Data Availability

The data that support the findings of this study are available from the corresponding author, HM, upon reasonable request. However, due to data governance and legal restriction, data cannot be shared without formal approval from appropriate authorities.
